# Atmospheric correction of vegetation reflectance with simulation-trained deep learning for ground-based hyperspectral remote sensing

**DOI:** 10.1186/s13007-023-01046-6

**Published:** 2023-07-29

**Authors:** Farid Qamar, Gregory Dobler

**Affiliations:** 1 Department of Civil and Environmental Engineering, University of Delaware, Newark, DE 19716 USA; 2 Data Science Institute, University of Delaware, Newark, DE 19716 USA; 3 Biden School of Public Policy and Administration, University of Delaware, Newark, DE 19716 USA; 4 Department of Physics and Astronomy, University of Delaware, Newark, DE 19716 USA; 5 Center for Urban Science and Progress, New York University, New York, NY 10003 USA

## Abstract

**Background:**

Vegetation spectral reflectance obtained with hyperspectral imaging (HSI) offer non-invasive means for the non-destructive study of their physiological status. The light intensity at visible and near-infrared wavelengths (VNIR, 0.4–1.0µm) captured by the sensor are composed of mixtures of spectral components that include the vegetation reflectance, atmospheric attenuation, top-of-atmosphere solar irradiance, and sensor artifacts. Common methods for the extraction of spectral reflectance from the at-sensor spectral radiance offer a trade-off between explicit knowledge of atmospheric conditions and concentrations, computational efficiency, and prediction accuracy, and are generally geared towards nadir pointing platforms. Therefore, a method is needed for the accurate extraction of vegetation reflectance from spectral radiance captured by ground-based remote sensors with a side-facing orientation towards the target, and a lack of knowledge of the atmospheric parameters.

**Results:**

We propose a framework for obtaining the vegetation spectral reflectance from at-sensor spectral radiance, which relies on a time-dependent Encoder-Decoder Convolutional Neural Network trained and tested using simulated spectra generated from radiative transfer modeling. Simulated at-sensor spectral radiance are produced from combining 1440 unique simulated solar angles and atmospheric absorption profiles, and 1000 different spectral reflectance curves of vegetation with various health indicator values, together with sensor artifacts. Creating an ensemble of 10 models, each trained and tested on a separate 10% of the dataset, results in the prediction of the vegetation spectral reflectance with a testing r^2^ of 98.1% (±0.4). This method produces consistently high performance with accuracies >90% for spectra with resolutions as low as 40 channels in VNIR each with 40 nm full width at half maximum (FWHM) and greater, and remains viable with accuracies >80% down to a resolution of 10 channels with 60 nm FWHM. When applied to real sensor obtained spectral radiance data, the predicted spectral reflectance curves showed general agreement and consistency with those corrected by the Compound Ratio method.

**Conclusions:**

We propose a method that allows for the accurate estimation of the vegetation spectral reflectance from ground-based HSI platforms with sufficient spectral resolution. It is capable of extracting the vegetation spectral reflectance at high accuracy in the absence of knowledge of the exact atmospheric compositions and conditions at time of capture, and the lack of available sensor-measured spectral radiance and their true ground-truth spectral reflectance profiles.

## Background

Hyperspectral imaging (HSI) provides a powerful non-invasive diagnostic tool for the near real-time study of the physiological status of vegetation. Plant biophysical characteristics, such as leaf and tissue structure, and biochemical features, such as pigments and water content, drive the interactions between incoming light (irradiation) and the leaves. Therefore, light reflected off of leaves carries information about the physiological and morphological properties of the target vegetation with which irradiation interacted [1]. Diseases and stress produce physiological changes in the plants’ metabolism, and can vary significantly in impact with the type of plant and cause of stress, including type of pathogen [2]. These changes to the vegetation’s characteristics include alterations to the biophysical and biochemical features that interact with light. Thus, capturing the vegetation spectral reflectance, particularly in the visible and near-infrared (VNIR, 0.4–1.0µm) wavelength range, provides information about the plant’s condition. Studies over the past two decades have utilized hyperspectral imaging to capture detailed information about the spectral characteristics of plants, and use them for a variety of investigations including the early detection of disease symptoms and pest infestations [3–5], and monitoring stress conditions and nutrient deficiency [6–8].

The attainment of vegetation spectral reflectance for the performance of such investigations is generally done using hyperspectral imaging sensors either proximally, such as by using flux towers [9, 10], or remotely using satellite [11, 12], aircraft [13, 14], uncrewed aerial vehicle (UAV) [15, 16], or ground-based platforms [17–19]. Regardless of platform, remotely obtained spectral radiances are heavily impacted by atmospheric effects, such as absorption and scattering from the presence of various gases and aerosols, and highly dependent on atmospheric conditions such as temperature, humidity, and solar angle. Additionally, the incoming solar spectral irradiance inherently contains wavelength dependent intensities, which interact with the atmosphere en route to the target vegetation, resulting in further modifications to the down-welling spectral irradiance prior to encountering the plant. Following the interaction between the down-welling irradiance and the vegetation, the reflectance of the vegetation impart additional changes to the spectrum. Prior to reaching the sensor, the light is also impacted by the atmosphere in the line of sight between the sensor and the target vegetation, where the impact magnitude can vary significantly depending on the platform and its distance to the target. Therefore, while the sensor-obtained spectral radiance contains information regarding the target vegetation, its heavy mixing with atmospheric and solar effects significantly impacts the quality of the extracted information, and its applicability to vegetation health inference in the presence of highly covariant variables such as atmospheric conditions and compositions [20, 21].

The spectral radiance reaching the sensor ($L_\lambda$) at wavelength $\lambda$ can be expressed mathematically using the radiative transfer equation:

1 $$\begin{aligned} \begin{aligned} {L_{\lambda } }&{= \{ E_{s\lambda } \cos {\sigma } \frac{R_\lambda }{\pi } T_{s\lambda } + \varepsilon (\lambda ) L_{\tau \lambda } + [F(L_{ds\lambda } + L_{d\epsilon \lambda })} \\&\quad {+ (1-F)(L_{bs\lambda } + L_{b\epsilon \lambda })]\cdot R_\lambda \} \cdot T_{\lambda } + L_{us\lambda } + L_{u\epsilon \lambda }} \end{aligned} \end{aligned}$$

where $E_{s\lambda }$ is the extraterrestrial solar irradiance, $\sigma$ is the incident angle of the solar irradiance, $T_{s\lambda }$ is the atmospheric transmission on the sun-target path, while $T_{\lambda }$ is the atmospheric transmission on the target-sensor path, $R_\lambda$ is the spectral reflectance of the target object, $\varepsilon$ is the spectral emittance of the target object, $L_{\tau \lambda }$ is the spectral radiance of a blackbody with temperature $\tau$, *F* is the shape factor denoting the fraction of the hemisphere that is obscured by background objects, $L_{ds\lambda }$ is the solar down-welling spectral radiance, $L_{d\epsilon \lambda }$ is the atmospheric down-welling spectral radiance, $L_{bs\lambda }$ is the solar reflected background radiance, $L_{b\epsilon \lambda }$ is the spectral radiance from the background due to self-emission, $L_{us\lambda }$ is the up-welling solar radiance, and $L_{u\epsilon \lambda }$ is the up-welling radiance due to self-emission. By assuming the object is located in an open area ($F = 1$), and since $L_{\tau \lambda }$ contributes less than 0.1% of the magnitude of the spectral radiance and can be considered negligible, the effective radiance at the sensor can be approximated and simplified in the VNIR, as shown in [22], to become:

2 $$\begin{aligned} {L_{\lambda } = E_\lambda \cdot T_\lambda \cdot R_\lambda + L_{us\lambda }} \end{aligned}$$

where $E_\lambda = [E_{s\lambda } \frac{\cos {\sigma }}{\pi } T_{s\lambda } + L_{ds\lambda } + L_{d\epsilon \lambda }]$ describes the total down-welling radiance incident on the target.

 To study the properties of vegetation using hyperspectral remote sensing, the at-sensor spectral radiance ($L_\lambda$) must be corrected to remove the atmospheric and solar effects ($E_\lambda$, $T_\lambda$, and $L_{us\lambda }$) and extract the reflectance spectrum ($R_\lambda$). Algorithmic methods used for the solar and atmospheric correction of HSI data can be grouped into two broad categories: scene-based empirical approaches, and radiative transfer approaches [23]. Scene-based empirical approaches, such as the Quick Atmospheric Correction (QUAC) [24], flat-field (FF) correction [25], and the Compound Ratio approach [21], rely on parameters being derived through prior knowledge or assumptions about in-scene elements. Such methods can be computationally efficient and accurate, but carry large uncertainties in the corrected spectral reflectance if insufficient information is obtained from the image, or when assumptions are idyllic [26]. On the other hand, radiative transfer approaches, such as the ATmospheric REMoval program (ATREM) [27], the Atmospheric CORection Now (ACORN) program [28], and Fast Line-of-sight Atmospheric Analysis of Spectral Hypercube (FLAASH) [29], rely on modeling the known physical mechanisms of interactions with radiation from first-principles in order to separate the atmospheric and solar effects from the target’s spectral reflectance. By modeling the propagation of light through an atmosphere of known parameters, these deterministic mechanisms can be highly accurate and precise. However, modeling radiative transfer from first-principles tends to be computationally expensive, and requires either the explicit knowledge of the atmospheric parameters, or assumptions and standard atmospheres that can create inaccuracies [30]. Alternatively, methods such as the inverse modeling of the captured spectral radiance allows for the constraining of the range of values that the atmospheric parameters can possess while exploiting the benefits of radiative transfer codes [31]. However, due to the significant covariance in the spectral impacts of solar and atmospheric parameters, methods that rely on high sampling of parameter space generally trade-off accuracy for computational efficiency.

To provide a mechanism for the accurate extraction of spectral reflectance in hyperspectral images without knowledge of the properties of in-scene elements or atmospheric parameters, deep machine learning models have been shown to provide end-to-end fully data driven methods that can efficiently exploit the abundance of information in the at-sensor spectral radiance, including the variable covariances [32]. Furthermore, deep learning models have been shown to be significantly adept at signal extraction in numerous applications beyond hyperspectral imaging and remote sensing. Such applications include wireless signal extraction [33–35], isolating sound and correcting audio distortion and interference [36–38], and the identification of cardiovascular disorders in the recording of the electrocardiogram (ECG) [39, 40]. The flexibility of deep neural networks, due to their large multi-parametric models and reliance on learning from large and comprehensive datasets, allows such methods to use little to no a priori knowledge to analyze noisy and mixed data to estimate the parameters of complex systems that range from astrophysics [41, 42] to quantum mechanics [43, 44]. In the domain of hyperspectral remote sensing, convolutional neural networks (CNNs) in particular, a subclass of neural networks that can generate associations between spectral features through a sequence of learned filter shapes, have seen substantial use in various hyperspectral analysis tasks over the past few years, including plant health and disease identification [45, 46], and plant speciation [47, 48], where it has been found to outperform comparable methods [49].

For the atmospheric correction of spectral reflectance in the longwave infrared (LWIR) wavelength range of 7.0–12 $\mu$m, [32] proposed a CNN composed of two parts: the encoder (Enc) and the decoder (Dec). The Enc-Dec CNN is trained and tested on synthetically produced spectral radiance for the known spectral reflectivity of several materials. Once training is complete, the model takes as input the observed at-sensor spectral radiance obtained at 8 viewing angles, and, as output, produces four spectral components of the radiative transfer equation (RTE) per viewing angle, which when combined using the RTE generates the atmospherically corrected spectral reflectance and associated uncertainty. This Enc-Dec CNN is further adapted by [50] to perform atmospheric correction on spectral reflectance in the visible and shortwave infrared wavelength range of 0.4$-$3.0 $\mu$m with the addition of a time-dependent component to the deep neural network’s architecture. In this modification, the dimensionally-reduced encoding of the at-sensor spectral radiance (output by the encoder) is concatenated with tensor representations of two temporal factors: the day and time of capture, prior to proceeding to the latent space and decoder to generate the RTE components. To increase the usability of this model in hyperspectral analysis, [50] train the model on the spectral reflectance of 42 MODTRAN built-in materials [51], 15 of which are vegetation spectra, and achieving an average accuracy of 95.72% at estimating atmospherically corrected spectral reflectance.

Remote sensing applications in the literature tend to be dominated by satellite, aerial, and UAV platforms, which generally have a down-looking (nadir) pointing towards the target. Therefore, atmospheric correction methods are often geared towards containing scientific modeling sufficient to contain the range of potential means that can affect spectral radiance arriving at sensors with viewing angles on the order of 90$^{\circ }$. Light reflecting off of a target object encounters atmospheres that substantially differ in composition, density, and length if travelling in the azimuth direction through to the top of the atmosphere and beyond, as opposed to travelling horizontally along the troposphere for up to several kilometers. The recent utilization of side-facing HSI platforms for the study of vegetation [18, 21], demonstrates that there is interest in the development of methods for the atmospheric correction of spectral reflectance for ground-based platforms, including for cases in which precise atmospheric parameters at time of capture are unknown.

In this paper, we present a method for the accurate estimation of vegetation spectral reflectance in the VNIR wavelength range from spectral radiance collected by ground-based remote HSI sensors. For this purpose, we produce numerous simulated unique at-sensor spectral radiances blended in various combinations with different simulated vegetation spectral reflectances, both generated using specialized radiative transfer simulation codes. We use the large sample of spectral radiance and associated spectral reflectance to train, validate, and test an ensemble of 10 separate time-dependent Enc-Dec CNN models that take the at-sensor spectral radiance as input, and output the estimated vegetation spectral reflectance, which is of equal spectral resolution and sampling as the input spectral radiance. We test and evaluate the transferability of the model by measuring its capability of accurately predicting the spectral reflectance from spectral radiance examples for which the network has not encountered either the atmospheric composition or the vegetation spectral reflectance in the training process. We then examine the limits in spectral resolution for which this proposed method remains reasonably viable, by repeating the training and testing process using systematically decreased spectral resolutions for the simulated spectra.

## Methods

**Fig. 1:**
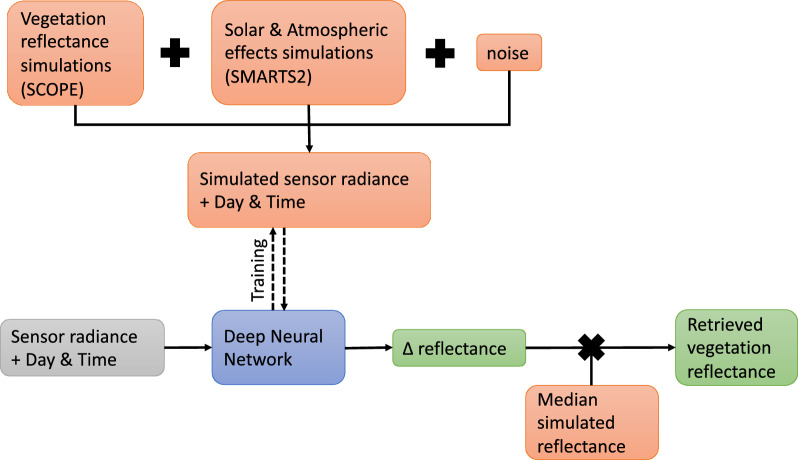
A graphic workflow of the time-dependent deep learning solution to extract vegetation spectral reflectance from remote hyperspectral imaging using simulated at-sensor spectral radiance

The framework for the retrieval of vegetation spectral reflectance from at-sensor spectral radiance obtained using ground-based remote shortwave (VNIR) hyperspectral imaging is summarized in Fig. 1. At the core of this framework is a time-dependent Enc-Dec CNN, with an architecture designed for extracting vegetation spectra from ground-based remote hyperspectral images. For this purpose, we use a neural network that is trained and tested using large and comprehensive samples of simulated vegetation spectral reflectance and solar and atmospheric attenuation effects. This neural network, once trained, takes as input an uncorrected at-sensor spectral radiance of target vegetation obtained with a ground-based remote HSI sensor, together with its capture day and time, and outputs the predicted deviation of the vegetation’s spectral reflectance ($\Delta$reflectance), calculated as a ratio from the median reflectance of all simulated vegetation spectra. The output is then converted into a spectral reflectance curve using the known median simulated vegetation spectral reflectance.

### Case study

This work is aimed at providing a method for the extraction of vegetation spectral reflectance from ground-based remote hyperspectral imaging. In particular, it targets imaging that was performed in the absence of knowledge of the exact atmospheric parameters at times of capture, and lack of known examples of concurrent at-sensor spectral radiance and vegetation spectral reflectance. Since this method requires the production of a large sample of simulated spectra whose parameters are tuned to resemble the properties of the hyperspectral sensor and the sensor-target geometry to train the deep neural network, it is most applicable to persistent and static remote sensing applications performed over extended periods of time. For such an application, the obtained spectral reflectance can be utilized for the study of the relative temporal changes in the status of vegetation.

**Fig. 2:**
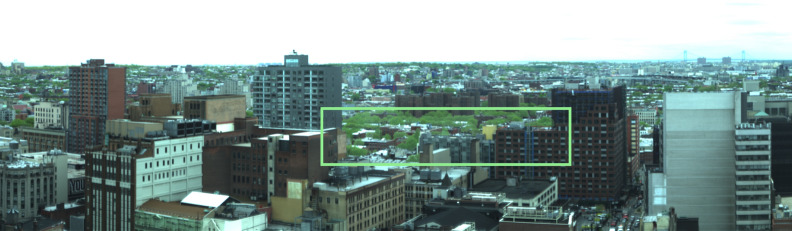
A false-color RGB (0.61 $\mu$m, 0.54 $\mu$m, and 0.48 $\mu$m) image of the scene of Downtown and North Brooklyn obtained by the Urban Observatory’s hyperspectral imaging system. In the green box are the highlighted vegetation pixels relevant to this work, which are located at roughly 1 km south of the sensor that is sited atop a 120 m tall building

To demonstrate a potential case to which this method can be applied to extract vegetation spectral reflectance from at-sensor spectral radiance, the VNIR hyperspectral imaging data collected by the “Urban Observatory” (UO) [19] facility in New York City (NYC) is selected as a case study. The instrument used in this work is a Specim Ltd. ImSpector V10E VNIR hyperspectral imager, a single slit scanning spectrograph with 1600 vertical pixels, capable of providing a spectral resolution of 1 nm in the 0.4–1.0µm wavelength range. The instrument is placed atop a 120 m ($\sim$400 ft) building in Brooklyn, facing south and horizontally aligned. To provide push-broom scanning, the instrument is mounted on a FLIR pan/tilt unit, allowing for each scan to have a field of view that is roughly $75^\circ \times 35^\circ$ with $1600\times 1600$ pixels and pixel axis ratio of $\sim 0.45$. The observations were carried daily during the month of May 2016, where persistent scans of the same scene were obtained at 15 min intervals during the day between 08h00 and 18h00. Figure 2 shows a composite RGB image of the scene (mapped to 0.61 $\mu$m, 0.54 $\mu$m, and 0.48 $\mu$m), with the vegetation patch of interest to this work highlighted. Given the geometrical setup of the scene, the spatial resolution of each of the vegetation pixels, which are located at roughly 1 km south of the sensor, is approximately 1 m$\times$0.45 m.

### Simulations

Combining the output of radiative transfer software that simulates a wide range of vegetation spectral reflectance given different plant health indicators, together with the output of software that simulates the impact of various solar and atmospheric conditions on the spectral reflectance of an object provided different atmospheric parameters and times of day and day of the year, results in obtaining simulated examples of possible spectral radiance with their associated spectral reflectance known. Creating a sufficiently comprehensive sample of simulated atmospheric effects and simulated vegetation spectral reflectrance allows a deep learning network to be trained, validated, and tested to extract the spectral reflectance of vegetation from the at-sensor spectral radiance.

#### Vegetation spectral reflectance

For obtaining a sufficiently representative sample of vegetation spectral reflectance for plants under various potential conditions, we rely on the Soil Canopy Observation of Photosynthesis and Energy fluxes (SCOPE) model to produce the required simulations. SCOPE provides simulations of the hyperspectral radiance and net radiation for vegetation by combining several radiative transfer models with a leaf biochemical model [52, 53]. Due to its coupling of photosynthetic, hydrological, and radiative transfer models, SCOPE has been used in a wide range of studies including the exploration of the relationship between fluorescence and photosynthesis [54–56], predicting evapotranspiration [57, 58], and productivity and yield monitoring [59]. With the goal of broad applicability, SCOPE models are based on physical principles and capable of modeling the spectral reflectance of vegetation with varying morphological and physiological properties. Furthermore, the radiative transfer routines of SCOPE are based on the SAIL model (Scattering by Arbitrarily Inclined Leaves) [60, 61], thus providing a vertically integrated radiative transfer and energy balance model capable of calculating the spectral reflectivity at the level of single leaves as well as the canopy level. The canopy representation in SCOPE is composed of various layers, in each the leaves can have different orientations, where the probability of the occurrence of each leaf zenith and azimuth are quantified using a leaf inclination distribution function (LIDF). Therefore, SCOPE is capable of differentiating the leaves by their orientation with respect to the sun as well as their vertical positions in the canopy to discriminate between sunlit and shaded leaves, and by exploiting the principle of linearity of the radiative transfer equation, the contributions of the shaded and sunlit leaves are vertically integrated to obtain the reflectivity of the canopy.

**Fig. 3:**
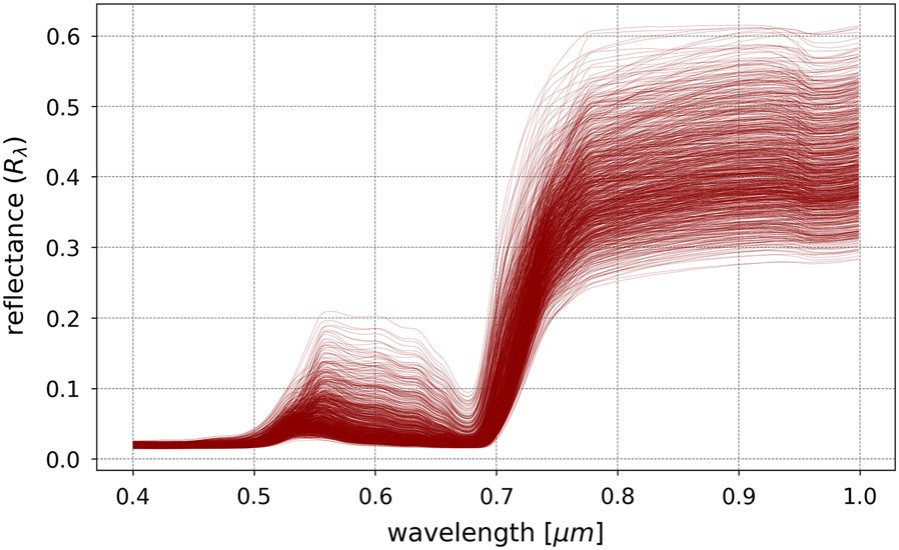
Vegetation spectral reflectance in the VNIR wavelength range simulated using SCOPE with varying morphological and physiological plant properties

To allow the deep neural network to encounter and learn from a wide range of potential spectra for the sake of model transferability, it is imperative to generate a sufficiently comprehensive sample of vegetation spectral reflectances. For this purpose, 1000 different spectra, shown in Fig. 3, were simulated using SCOPE in which each has a unique combination of the following parameters:

- Chlorophyll AB content, ranging between 5.0p µg/cm$^2$ and 80.0 µg/cm$^2$;
- Carotenoid content, ranging between 5.0 µg/cm and 20.0 µg/cm$^2$;
- Dry matter content, ranging between 0.0 g/cm$^2$ and 0.02 g/cm$^2$;
- Leaf water equivalent layer, ranging between 0.001 cm and 0.02 cm;
- Senescent material fraction, ranging between 0% and 50%;
- Leaf area index, ranging from 2.0 and 4.0;
- Leaf inclination distribution function parameters (LIDFa and LIDFb), ranging from –0.5 and 0.5.

#### Solar and atmospheric effects

To simulate the use of ground-based VNIR hyperspectral remote sensing, the SMARTS2: Simple Model of the Atmospheric Radiative Transfer of Sunshine [62, 63] software is used to calculate the at-sensor spectral radiance provided solar conditions, a synthetic atmospheric composition, and object reflectance. SMARTS2 is a relatively computationally efficient and comprehensive radiative transfer code written in Fortran77, capable of predicting the effect on the observed photon flux at the sensor from a given atmospheric composition, and a particular solar angle calculated given a user defined location (latitude and altitude), height, and pointing angle of the sensor. This code was developed using physical and mathematical principles including radiative transfer theory and atmospheric chemistry, with the intention of matching the output from rigorous radiative codes such as MODTRAN to within 2% [63], with a significantly lower calculation time (factor of 25) [28]. Numerous examples of its use as a means for simulating the solar and atmospheric impacts on radiation in narrowband hyperspectral imagery can be found in the remote sensing literature [31, 64, 65].

As a demonstration of the framework’s functionality, we utilize the experimental setup presented in the Case study section to assume a particular HSI camera, a location for the sensor and observed targets, and time and date of the obtained data. Using SMARTS2, we generate a comprehensive sample of atmospheric effects by simulating 1440 different atmospheres, each of which containing a unique combination of the following indicators:

- Relative humidity at site level, ranging between 1% and 99%;
- Precipitable water above the site altitude, ranging between 1.0 g/cm$^2$ and 12.0 g/cm$^2$;
- Hour of the day, ranging between 08h00 and 18h00;
- Day of the month of May, ranging between 1 and 30;
- Atmospheric temperature at site level, ranging from 5 °C to 30 °C to $30^{\circ }$C;
- Aerosol optical depth at 500 nm ($\tau _5$), ranging between 0.5 to 5;
- Oxygen (O$_2$) concentration, ranging from 21% to 22%;
- Ozone (O$_3^{Ab}$) total-column abundance (excluding tropospheric pollution), ranging from 0.2 to 0.5 atm-cm;
- Volumetric concentrations in the assumed 1-km deep tropospheric pollution layer for each of the following pollutants:
  - Tropospheric ozone (O$_3^{p}$) due to pollution, ranging from 0 to 0.02 ppmv;
  - Nitrous acid (HNO$_2$), ranging from 0 to 10.0 ppbv;
  - Nitrogen dioxide (NO$_2$), ranging from 0 to 0.2 ppmv;
  - Nitrogen trioxide (NO$_3$), ranging from 0 to 0.2 ppbv;
  - Sulfur dioxide (SO$_2$), ranging from 0 to 0.2 ppmv.

While SMARTS2 is capable of varying the concentrations of numerous other gases, including CH$_{2}$O, CH$_{4}$, CO, CO$_{2}$, HNO$_{3}$, NO, BrO, ClNO, N$_2$O, N$_2$, and NH$_3$, the absorption coefficients of these molecules are outside the VNIR range of wavelengths explored in this work. Since varying these parameters does not have an impact on the simulated atmospheric effects in the 0.4 – 1.0 $\mu$m wavelength range, they were therefore held constant for all simulations. Other user-defined parameters that did not vary throughout the generation of the simulations are:

- The extraterrestrial top-of-atmosphere solar spectral irradiance, which was set to the synthesized spectrum from [66];
- The latitude, longitude, and altitude, which were set to the location of the UO’s hyperspectral camera in NYC, the height of the building on which it was placed, and its tilt angle and surface azimuth of the setting as described in the Case study section;
- The aerosol model, which was set to a humidity-dependent urban aerosol model [67].

**Fig. 4:**
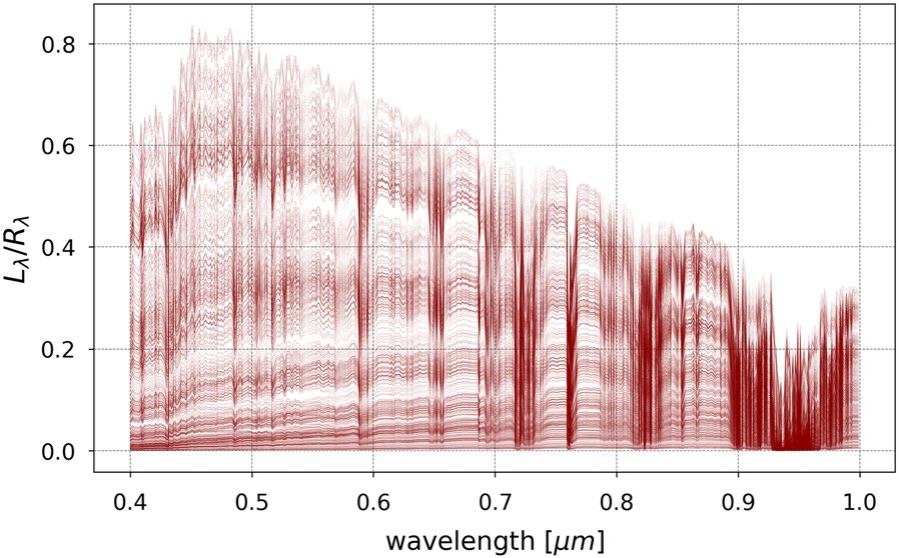
A sample of the 1440 SMARTS2 simulated at-sensor spectral radiances ($L_\lambda$) in the VNIR wavelength range for a single SCOPE simulated spectral reflectance ($R_\lambda$), divided by the reflectance to show the atmospheric and solar effects from varying their conditions for a static sensor with known location and pointing

A sample of 1440 unique at-sensor spectral radiances for each vegetation spectral reflectance obtained using SCOPE were generated using SMARTS2. Dividing the spectral radiances in Eq. (2) by the vegetation spectral reflectance ($R_\lambda$) produces the wavelength dependent spectral effects of each of the atmospheres and solar angles, which are visualized in Fig. 4. Multiplying a vegetation spectral reflectance spectrum ($R_\lambda$) from Fig. 3 obtained from SCOPE with the $L_\lambda /R_\lambda$ spectrum from Fig. 4 obtained from SMARTS2, with the addition of Gaussian-distributed random noise and sensor artifacts such as quantum efficiency, results in generating the radiance as measured by the sensor while retaining the “ground-truth” reflectance of the observed object.

### Encoder-decoder network

Convolutional neural networks are prevalent in the field of hyperspectral image analysis due to their effectiveness at tasks such as image segmentation [68–70] and pixel classification [71–73]. In recent years, CNNs have been utilized to develop deep learning models to address the problem of extracting the spectral reflectance from the spectral radiance. Such a problem requires a model capable of handling redundant information in both input and output, the inclusion of temporal factors, and producing an output vector of similar dimension to the input vector. Therefore, we opt to use a variation of the architecture introduced in [50], modified to enhance the accuracy of the extraction of the reflectance spectra, primarily by narrowing its scope of use to vegetation only.

**Fig. 5:**
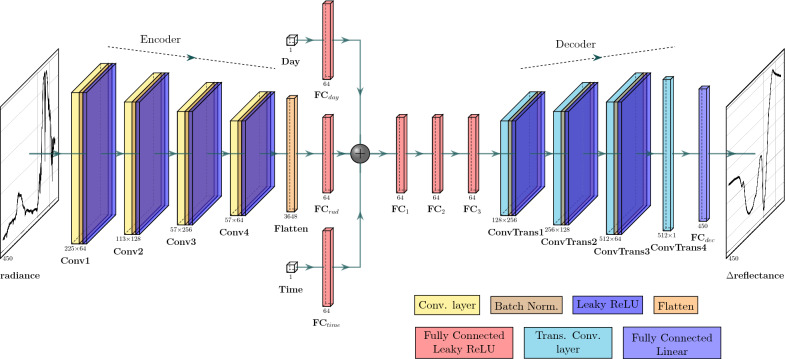
The architecture of the time-dependent encoder-decoder convolutional neural network (Enc-Dec CNN)

In Fig. 5, we show the architecture of our time-dependent encoder-decoder neural network (Enc-Dec CNN) for atmospheric correction of vegetation spectral reflectance obtained using ground-based remote hyperspectral sensors. The model relies on three main blocks, namely a convolutional encoder, fully encoded layers in latent space, and a convolutional decoder. The Enc-Dec CNN takes in as inputs the spectral radiance as observed by the sensor, together with capture time of the day and day of the year. The input spectrum is a min-max normalized spectral radiance curve, with a spectral sampling of 1 nm in the wavelength range of 0.4 $\mu$m to 0.85 $\mu$m (450 wavelength bands). This spectrum also includes sensor artifacts, such as noise and quantum efficiency.

Spectral information, particularly at high spectral resolutions, contains significant redundancies due to correlations between adjacent bands, and the presence of multiple absorption and emission features for individual gases and materials [74]. Therefore, the purpose of the encoder in our architecture is to reduce the spectral dimensionality and remove correlated and repetitive information from the input spectral radiance. The encoder is composed of four convolutional blocks, each consists of a convolutional layer, a batch normalization layer, and a Leaky Rectified Linear Unit (LeakyReLU) activation function. The hyperparameters for each layer, including numbers and sizes of convolutional filters, stride parameters, and output shapes, are listed in Table 1. Strided convolutional layers were selected for the encoder block rather than max pooling layers in order to reduce the input’s spectral dimensionality while maintaining necessary detailed information from the spectral radiance.

**Table 1:** Parameters of the time-dependent encoder-decoder convolutional neural network (Enc-Dec CNN)

Layer		Number of filters	Size of each filter	Stride	Output size
Input	Radiance	–	–	–	450 × 1
Encoder	Conv1	64	50	2	225 × 64
	Conv2	128	25	2	113 × 128
	Conv3	256	5	2	57 × 256
	Conv4	64	256	1	57 × 64
Fully connected layers	FC$_{rad}$	–	–	–	64 × 1
	FC$_{day}$	–	–	–	64 × 1
	FC$_{time}$	–	–	–	64 × 1
	FC$_1$	–	–	–	64 × 1
	FC$_2$	–	–	–	64 × 1
	FC$_3$	–	–	–	64 × 1
Decoder	ConvTrans1	256	5	2	128 × 256
	ConvTrans2	128	25	2	256 × 128
	ConvTrans3	64	50	2	512 × 64
	ConvTrans4	1	64	1	512 × 1
	FC$_{dec}$	–	–	–	450 × 1
Output	$\Delta$reflectance	–	–	–	450 × 1

To account and control for the crucial impact of diurnal and seasonal variations on vegetation spectral reflectance, solar angles, and the atmosphere, the day and time of capture are fed into individual fully connected layers. Concurrently, the output from the encoder is flattened into a 1D representative tensor and fed through a similar fully connected layer. Concatenating the encoded output of the spectral radiance with the outputs of the two independent fully connected layers produces the input for the latent space block. This block is composed of three fully connected layers with LeakyReLU activation functions and 64 neurons in each. The purpose of the latent space block is to summarize the input representative tensor (radiance, time, and day) into a tensor representative of the deviation of the vegetation spectral reflectance from that of the median simulated spectrum ($\Delta$reflectance).

Finally, the purpose of the decoder block is to expand and upsample the output representative tensor. Given that the spectral reflectance and spectral radiance share similar dimensions, the parameters of the four transposed convolutional layers in the decoder block are approximately the inverse of those in the encoder block, as shown in Table 1. The first three transposed convolutional layers are each followed by a combination of a batch normalization layer and LeakyReLU activation, while a fully connected layer with a linear activation follows the last layer to output the $\Delta$reflectance vector.

It is worth noting that due to the low variance among vegetation spectral reflectance, a machine learning algorithm can simply output the median spectral reflectance and be able to achieve $r^2\sim 90\%$. To avoid underfitting, we opt to utilize the wavelength-dependent deviation $\Delta$reflectance, calculated as a ratio of the spectral reflectance to the median simulated spectral reflectance, instead. This treatment produces output with greater variance among its members, which provide greater confidence in the $r^2$ score being representative of the model’s performance in learning to extract the output spectrum from the spectral radiance given capture time and day.

## Results

### Data preparation

The at-sensor spectral radiance is modeled by combining a SCOPE simulated vegetation spectral reflectance, a SMARTS2 simulated atmospheric absorption and solar effects spectrum, and sensor artifacts such as noise and quantum efficiency. Creating every possible unique combination of the 1000 simulated vegetation spectral reflectances and the 1440 solar and atmospheric effects spectra generates 1,440,000 unique $L_\lambda$ instances. These instances can then be split into training, testing, and validation sets to use with the machine learning model. In this treatment, while the instances are unique since each has a particular combination of vegetation, atmospheric, and solar parameters that solely belong to it, many instances share either a spectral reflectance or a spectral emission and transmission profile across sets.

**Fig. 6:**
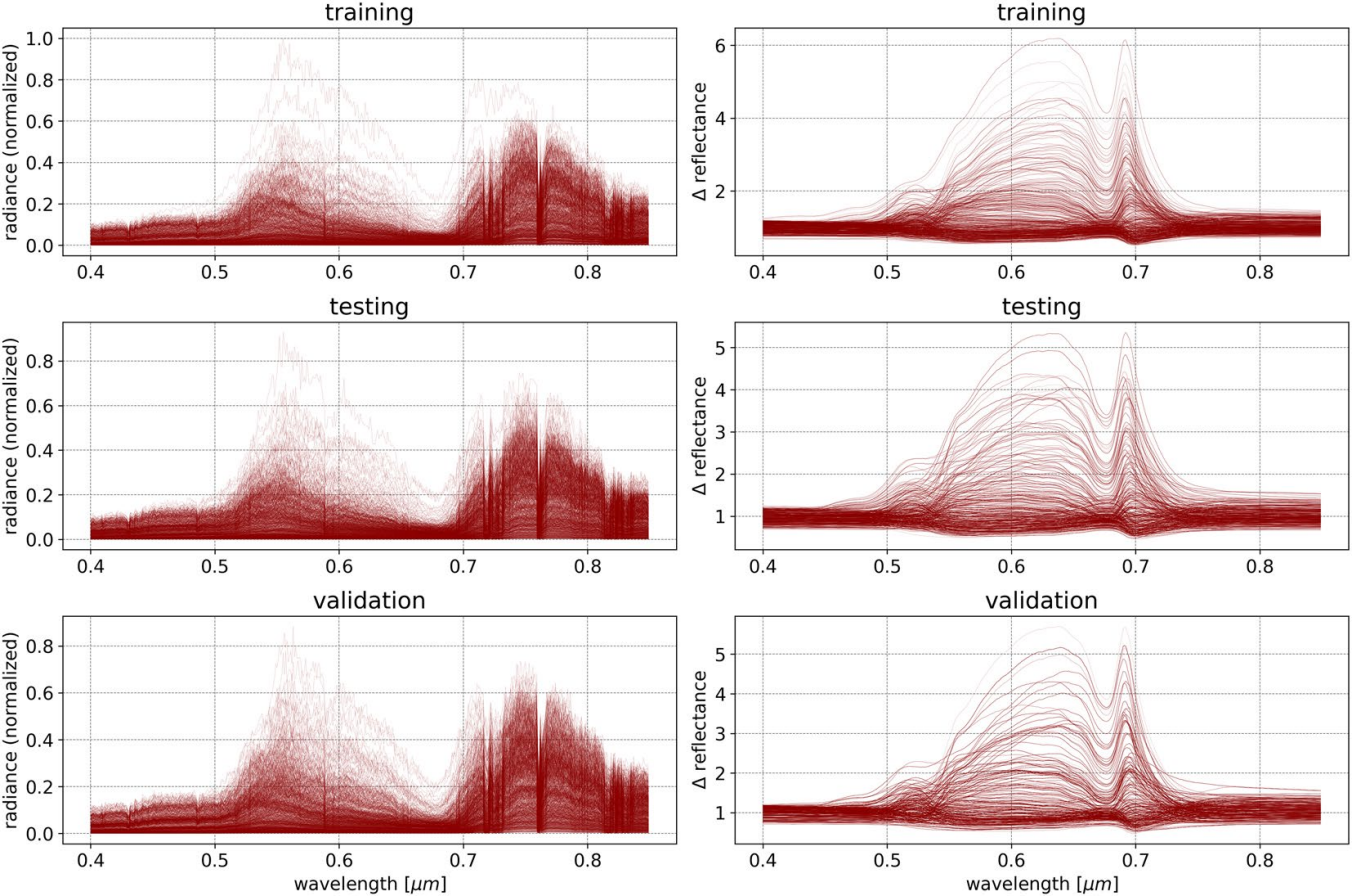
A subset of the normalized simulated at-sensor spectral radiance (*left*) from the training (*top*), testing (*middle*), and validation (*bottom*) sets, and their associated $\Delta$reflectance (deviation of spectral reflectance from the median, *right*). After splitting both reflectance and solar and atmosphere simulations into 3 independent sets (50% training, 30% testing, and 20% validation) each, we generate 360,000 training instances, 129,600 testing instances, and 57,600 validation instances. Each of the 3 sets are separated into 10 equal subsets, where each subset is used to train, validate, and test a neural network model independently for a total of 10 models

The purpose behind utilizing independent data sets in machine learning is to reduce overfitting, and assure the model performance metrics reflect its transferability to unseen instances in which neither spectral reflectance nor emission and transmission have been previously encountered by the model. Therefore, to assure independence between sets at the cost of a reduced total number of available instances, we randomly divide our simulated vegetation spectral reflectance into three sets: 50% training, 30% testing, and 20% validation. Similarly, we divide our simulated atmospheric and solar profiles into three sets of the same ratios as the spectral reflectance sets. The final sets are obtained by creating all possible combinations of spectral reflectance and solar and atmospheric effects within each set separately to produce 360,000 training instances, 129,600 testing instances, and 57,600 validation instances, which are shown in Fig. 6. Since this method is directed at hyperspectral analysis cases in which ground-truth spectral reflectances are unavailable, we exploit the large simulation sample size to increase the confidence in the model performance metrics being reflective of the accuracy when applied to real-world measurements. We randomly divide each of the training, testing, and validation dataset into 10 equal and separate subsets. Each subset is then independently used to train and test a neural network model, for a total of 10 models. This allows for the prediction of the final output using an ensemble of 10 independently trained and tested models, and provides an uncertainty range for the prediction and for the model’s overall accuracy.

The $\Delta$reflectance spectra shown in Fig. 6 are calculated as the deviation from the median simulated spectral reflectance. Each spectral radiance curve is generated by combining a vegetation spectral reflectance with a solar and atmospheric effects profile. To simulate the effects of the sensor as described in the Case study section, the resulting spectrum is multiplied by the sensor’s quantum efficiency curve. In this study we chose the Specim ImSpector V10E efficiency curve, which rises from slightly above 30% at 0.4 $\mu$m to approximately 60% at 0.6 $\mu$m, returning to slightly above 30% at 0.8 $\mu$m, and falling to less than 5% at 1.0 $\mu$m. This produces the photon flux curve (units of photons$\cdot$m$^{-2}\cdot$sr$^{-1}$), which can be converted into spectral radiance (units of W$\cdot$m$^{-2}\cdot$sr$^{-1}\cdot$nm$^{-1}$). Due to the presence of sensor noise in the recorded spectral radiance, a peak signal-to-noise ratio (SNR) of 20:1 is commonly assumed for HSI sensors [75–77]. Provided that the instrument in the case study utilizes a charge-coupled device (CCD) sensor to measure the incident photon flux and convert the signal from analog to digital output, various sources of noise, including photon noise, dark current, photo response nonuniformity, and read-out noise, contribute to the SNR. Modeling noise in hyperspectral imaging is a challenging task due to the presence of these multiple and varied noise sources, and remains an active topic of research [78, 79]. A common approach to noise modeling is to consider it as a mixture of two types: signal dependent noise, and signal independent noise [80]. Signal dependent noise, which includes photon (shot) noise, can be estimated by a Gaussian distribution where the noise variance is spectrally correlated [81]. Therefore, to account for this type of noise, a Gaussian distribution with a standard deviation of 5% of the spectral radiance at each wavelength channel is stochastically sampled, and added to the spectral radiance at each wavelength channel. On the other hand, signal independent noise, which includes sources such as thermal noise and quantization noise, are typically modeled by a simpler signal independent Gaussian additive noise [82, 83]. To include these types of noise, and provided that the spectral radiances were normalized to have values in the 0–1 range, a Gaussian distribution with a standard deviation of 0.05 was randomly sampled for each wavelength in each spectral radiance, and added to the spectral radiances.

### Training, validation, and testing

Each individual Enc-Dec CNN model presented here is trained using a unique set of 36,000 training examples. During the training, 5760 instances are used as validation examples, and following the training, 12,960 testing instances are used to measure the neural network’s performance and transferability. For the training, the $\ell ^2$-norm (Euclidean distance) is chosen to measure the distance between the network’s output and $\Delta$reflectance ground-truth. To optimize the parameters in the model while minimizing the objective function, the Adaptive Moment Estimation (ADAM) optimizer [84] is selected with an initial learning rate of $1\times 10^{-4}$, and the mean squared error (MSE) as the loss function. At each epoch, to reduce the memory usage and contribute to reducing overfitting, a batch size of 100 training instances is used in each forward and backward pass in the backpropagation process. The training is allowed to run up to 500 epochs, with an early stopping mechanism implemented to monitor the validation loss, and stop the learning process if 20 epochs pass without a sufficient change in validation loss. Once stopped, the parameter weights are brought back to the values they were assigned at the best performing epoch in the callback history.

When a neural network’s training is complete, 12,960 unseen testing instances are used to evaluate the trained model’s capability in predicting the vegetation spectral reflectance given the at-sensor spectral radiance and time and day of image capture. Table 2 shows a summary of the evaluation metrics for each of the three independent sets: training, validation, and testing, presented as the median of 10 independent models and their standard deviation (1$\sigma$) uncertainty. The similarity in all evaluation metrics between the three sets provides assurance that overfitting can be excluded from consideration as an influential factor in this study. Furthermore, the performance on the training set provides an indication of the model’s transferability to unseen examples of either reflectance, atmospheric absorption and scattering, solar angles, or all simultaneously. Here, the testing set in total shows a coefficient of determination $r^2=92.0\%(\pm 1.0)$ between the actual and predicted $\Delta$reflectance values at all wavelengths.

**Table 2:** Model evaluation on the training, validation, and testing sets, showing the median and 1$\sigma$ uncertainty for $r^2$, root-mean-square error (RMSE), mean absolute error (MAE), and mean absolute percentage error (MAPE)

Data set	$r^2$ [%]	RMSE	MAE	MAPE
Training	94.0$^{\pm 1.0}$	0.107$^{\pm 0.009}$	0.089$^{\pm 0.009}$	7.3$^{\pm 0.6}$
Validation	92.9$^{\pm 1.1}$	0.128$^{\pm 0.008}$	0.105$^{\pm 0.009}$	8.3$^{\pm 0.7}$
Testing	92.0$^{\pm 1.0}$	0.134$^{\pm 0.009}$	0.105$^{\pm 0.009}$	8.3$^{\pm 0.7}$

**Fig. 7:**
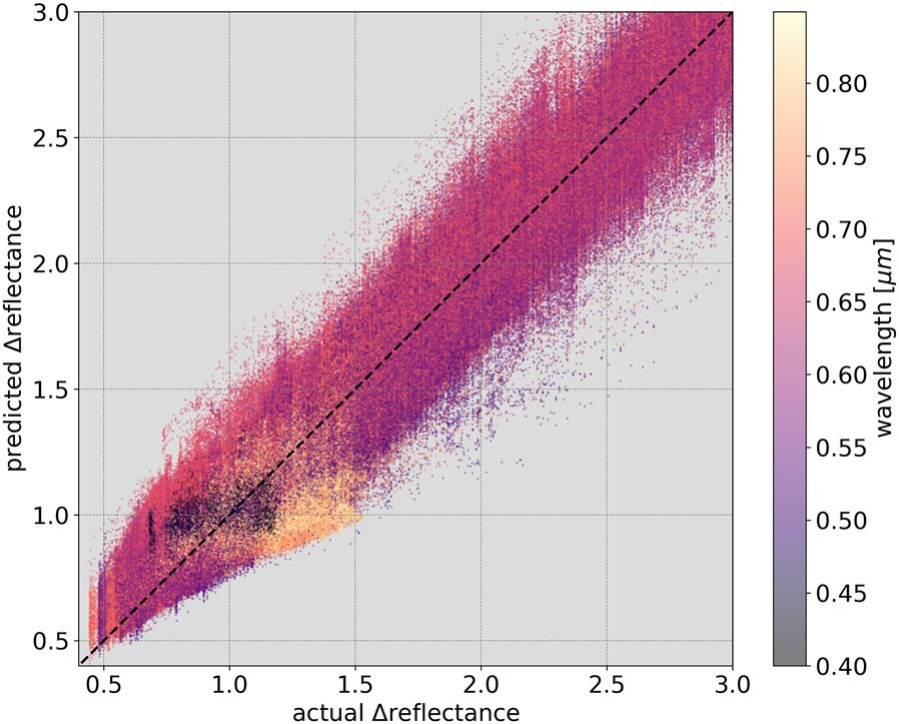
The actual $\Delta$reflectance of the simulated spectra in the testing set at each wavelength against model predicted values. The black dashed diagonal line has a slope of 1 and shows the case of perfect agreement between predicted and actual values. Overall, the models show $r^2=92.0\% (\pm 1.0)$ between the actual and predicted values

**Fig. 8:**
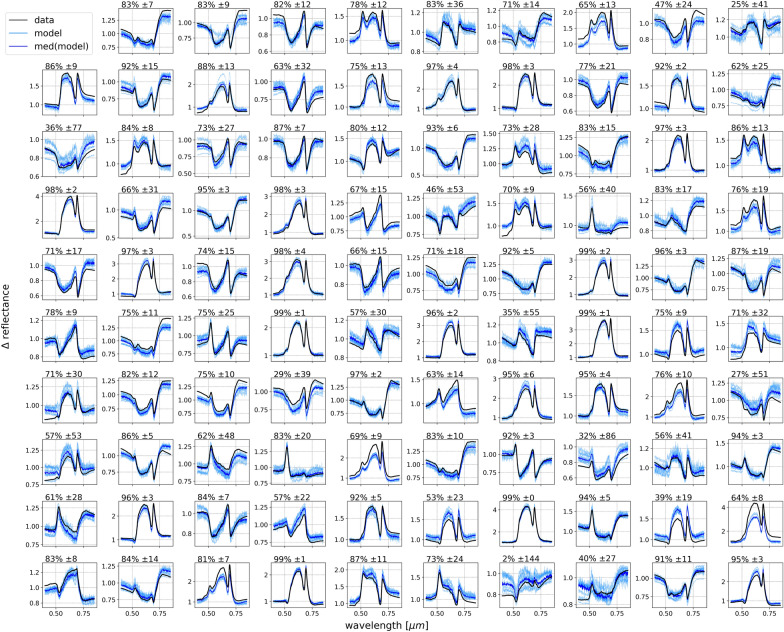
A randomly chosen sample of 99 spectra from the testing set, showing the actual simulated deviation from the median reflectance spectrum, $\Delta$reflectance, in *black*, each of the 10 Enc-Dec CNN model predicted $\Delta$reflectance spectra in *light blue*, and the median predicted model in *blue*. The median $r^2$ and associated 1$\sigma$ uncertainty from all models are listed for each instance at the top of each plot

To examine the factors contributing to this high $r^2$, Fig. 7 shows the predicted $\Delta$reflectance against the actual simulated $\Delta$reflectance at all wavelengths for all testing instances. The high testing score is evidently the product of high accuracy (inferred from the dense distribution of points about the diagonal where the predicted and actual values are similar), and high precision (from the consistent and low error variance, as demonstrated by the spread of values around the diagonal). This can be qualitatively observed in Fig. 8, which shows 99 randomly selected example instances of the actual wavelength-dependent $\Delta$reflectance spectra against predicted spectra from all 10 models and their median curve. The majority of instances show high correlation and similarity between the actual $\Delta$reflectance and the model prediction, with relatively high agreement among the individual models.

**Fig. 9:**
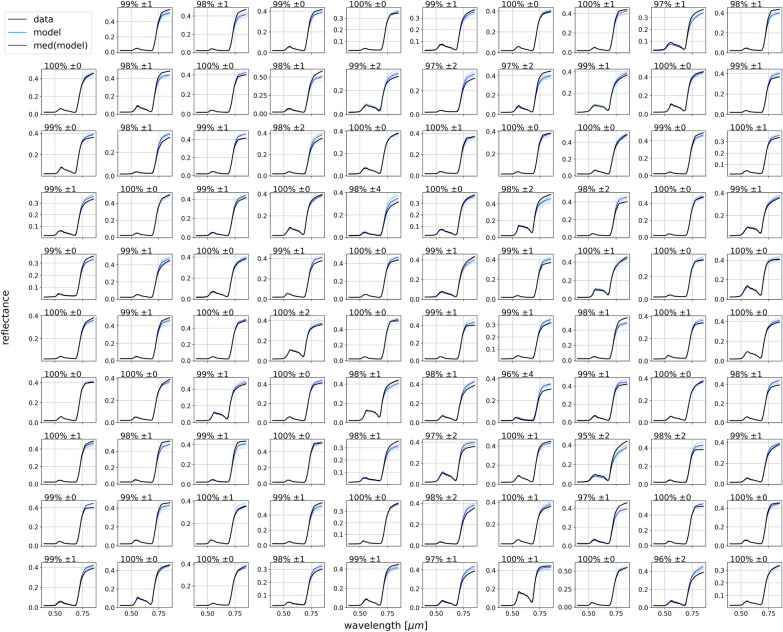
A randomly chosen sample of 99 spectral reflectance curves from the testing set, showing the actual simulated spectral reflectance in *black*, each of the Enc-Dec CNN model predicted spectral reflectance in *light blue*, obtained by multiplying the model’s output $\Delta$reflectance by the median simulated spectral reflectance and convolving the result using the FWHM of 6 nm. The median model predicted spectral reflectance is shown in *blue*, and the median $r^2$ and associated 1$\sigma$ uncertainty from all models are listed at the top of each plot

To assess the capacity of the proposed framework at accurately estimating the vegetation spectral reflectance from the at-sensor spectral radiance and two temporal factors (time and day), we must first obtain the model-predicted spectral reflectance. Using the known vegetation median spectral reflectance, the model-predicted $\Delta$reflectances are converted into reflectances. These spectral reflectances are smoothed by convolving with a Gaussian filter that has a standard deviation equal to the full width at half maximum (FWHM) of the sensor (6 nm), while maintaining the spectral resolution of the output spectral reflectance at 1 nm channels. Comparing the final, smoothed, model-predicted spectral reflectance with their ground-truth, simulated vegetation spectral reflectance yields a coefficient of determination $r^2=98.1\% (\pm 0.4)$. This high accuracy is qualitatively evident in Fig. 9, which shows a sample of 99 randomly selected testing instances that compare the model predictions with their respective spectral reflectance ground-truths. The vast majority of instances show prediction results indistinguishable from the ground-truth with $r^2 = 100\%$, the remaining instances show $r^2 > 96\%$, with uncertainties that rarely exceed 1%.

**Fig. 10:**
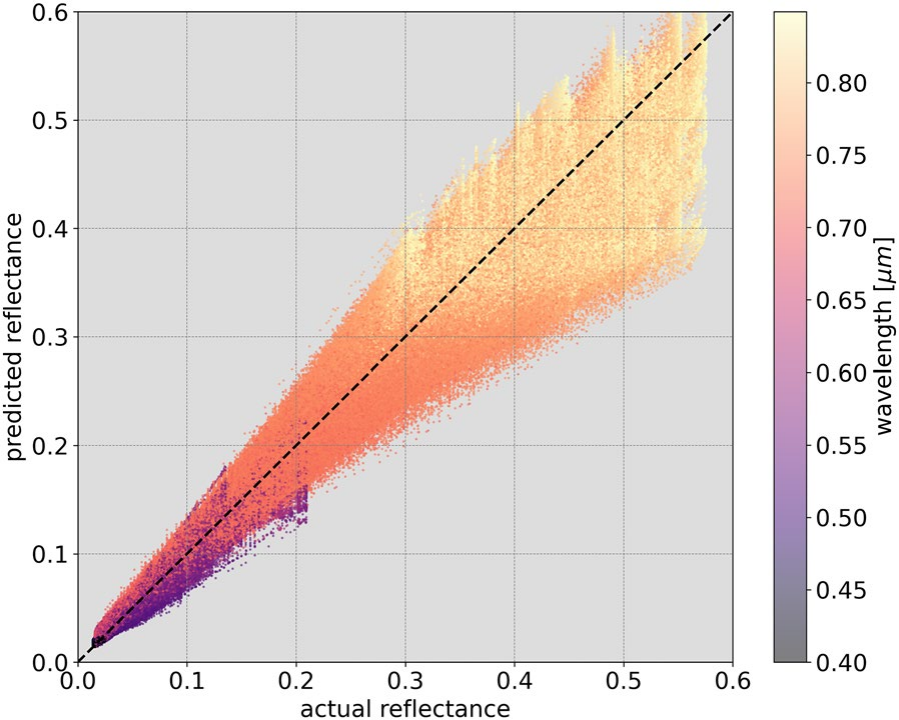
The actual spectral reflectance of the simulated spectra in the testing set at each wavelength against the model predicted values. The black dashed diagonal line has a slope of 1 and shows the case of perfect agreement between predicted and actual values. The model shows $r^2=98.1\% (\pm 0.4)$ between the actual and predicted values. In contrast with Fig. 7, vegetation spectral reflectance generally exhibit greater reflectance at longer wavelengths, as is expected beyond the red edge ($\sim 0.7\mu$m)

In Fig. 7, it can be noted that instances belonging to longer wavelengths appear to have a large concentration of points with actual and predicted $\Delta$reflectance values around 1. On the other hand, instances belonging to shorter wavelengths are more apparent at the extremes of the $\Delta$reflectance distribution. This indicates that the spectral reflectance in general shows relatively little variability from the median at longer wavelengths than at shorter wavelengths. This deviation from the median at short wavelengths could be explained as the product of Mei and Rayleigh scattering [85], which refer primarily to the elastic scattering of light from atomic and molecular particles with diameter ranging from approximately one-tenth to slightly larger than the wavelength of the incident light. Provided that aerosol optical depth was used in SMARTS2 to produce simulations with aerosol conditions that vary from the clear to the extremely polluted atmospheres in an urban setting, it is expected that such variations from the median in the $\Delta$reflectance spectra are primarily the products of scattering of light from aerosols. From Rayleigh’s law, it can be inferred that scattering at shorter wavelengths in the visible spectrum ($\sim$0.4 $\mu$m) is higher by a factor of $\sim$10 compared with that at longer wavelengths ($\sim$0.7 $\mu$m). However, in Fig. 9 we see that in the examples in which there is misalignment between actual and predicted spectral reflectance, the disagreement is visible only at longer wavelengths. Vegetation spectral reflectance exhibit similar general profiles across species, health status, etc., primarily due to chlorophyll and photosynthesis. As shown in Fig. 10, all instances belonging to shorter wavelengths have low reflectance values in comparison with instances belonging to longer wavelengths. Although the spectral reflectances show relatively greater variance at shorter wavelengths, due to the low magnitude of the reflectance, the model is capable of predicting the spectral reflectance at lower wavelengths with greater accuracy than at greater wavelengths. The distribution of points around the diagonal is compact and dense for instances belonging to wavelengths that range from $0.4\mu$m to $\sim 0.75\mu$m, beyond which the diffusion of points markedly diverges away from the diagonal.

### Spectral resolution

A wide range of remote sensing devices are used to uncover information on the status of vegetation from the properties of their spectral reflectance at various wavelengths. Some spectrographs offer a high characteristic spectral resolution, such as the Specim ImSpector V10E with a possible spectral resolution of 1 nm, to allow the user to obtain a wide range of detailed information regarding the vegetation and the atmosphere, including redundant information. Other spectrographs focus on capturing spectral reflectance only at required wavelengths to be used in calculating specific vegetation indices, such as the Apogee Instruments, Inc.’s Normalized Difference Vegetation Index (NDVI) sensor that captures light intensity in only 2 spectral bands—650 nm and 810 nm—with 65 nm FWHM each. Therefore, we examine the capability of the proposed framework at estimating the vegetation $\Delta$reflectance from the spectral radiance obtained with sensors that have spectral resolutions ranging proportionally from high (450 channels between 0.4 $\mu$m and 0.85 $\mu$m, with 1 nm FWHM), to low (2 NDVI channels, with 65 nm FWHM).

**Fig. 11:**
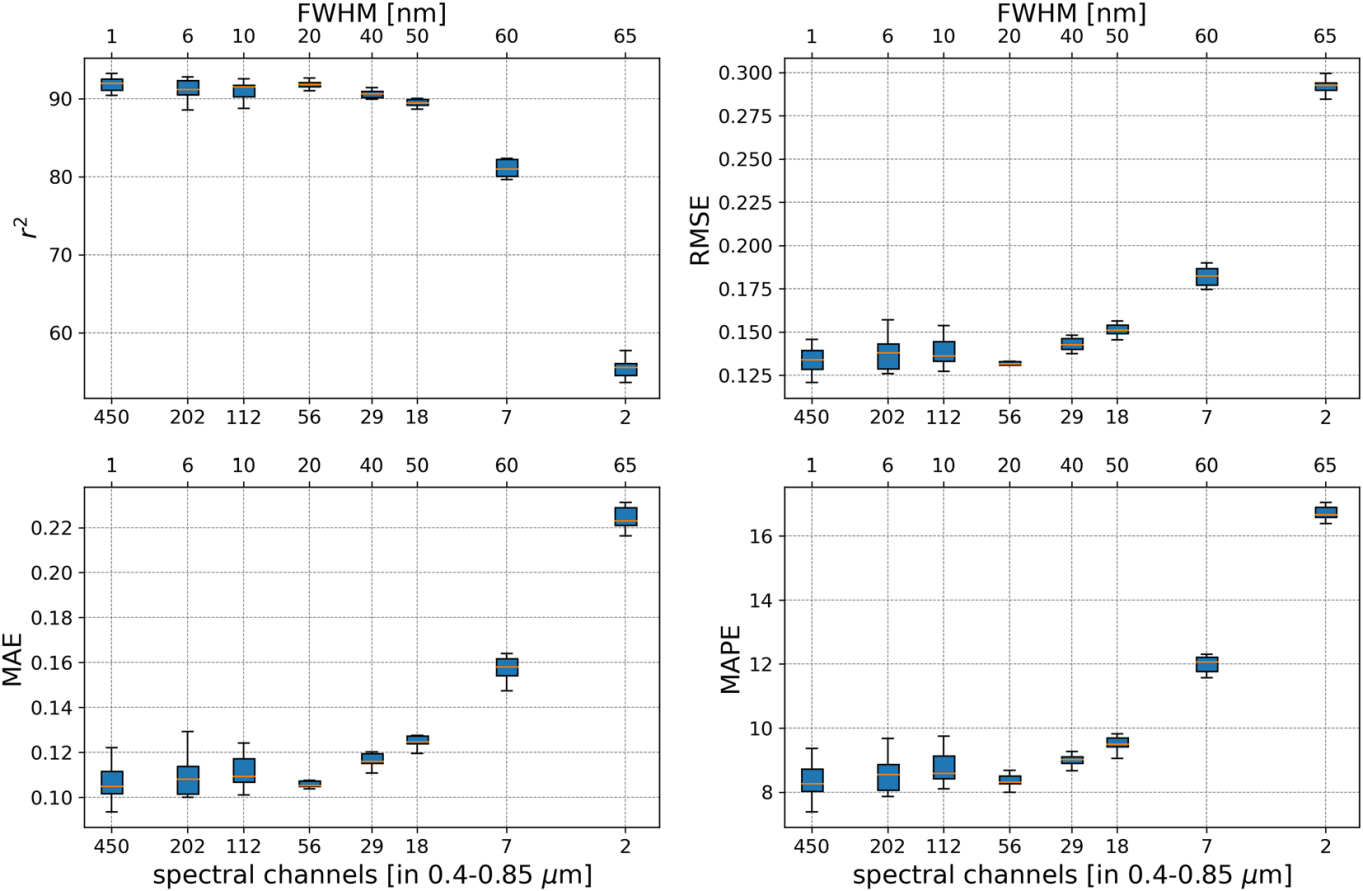
Performance metrics of the testing set for models trained and tested with spectral radiance and reflectance at systematically reduced spectral resolution and increased FWHM. Noting the range of the spectral channels on the x-axis are presented in logarithmic scale, the model’s accuracy remains consistently $>90\%$ down to a resolution of 29 channels (width of 15.5 nm). Accuracy lower than 80% occurs only at the lowest possible resolution of 2 channels

Individual and independent Enc-Dec CNN models with the architecture presented in Fig. 5 are trained, validated, and tested using vegetation spectral reflectance of systematically reduced spectral resolution. CNN models applied to spectral analysis are known to be sensitive to the filter sizes in their convolutional layers [72]. Therefore, in each model the number of filters and their kernel sizes were reduced in proportion to the reduction in spectral resolution in order to maintain an equivalent spectral filter across models. The metrics from the comparison of each model’s predicted $\Delta$reflectance for its respective testing set with the ground-truth, are presented in Fig. 11 for all models. For spectra with the highest resolutions starting at 1 nm FWHM (450 bands in 0.4–0.85 µm, or 600 channels in VNIR) down to 40 nm FWHM (29 bands in 0.4–0.85 µm, or 40 channels in VNIR), the model was consistently and equally capable of extracting the vegetation $\Delta$reflectance, with $r^2 > 90\%$ within uncertainty. Following which, a decrease in $r^2$ with decreasing spectral resolution becomes apparent, however, $r^2$ remains within the range of 80% for all but the lowest possible spectral resolution of two 65 nm wide channels.

### Application to case study

The method for solar and atmospheric correction of vegetation spectral reflectance presented in this work is aimed at ground-based, remote hyperspectral imaging in the VNIR wavelength range, for which the detailed and synchronous knowledge of atmospheric and solar parameters are unknown. Provided that this method relies on the generation of a large sample of simulated spectral reflectance and spectral radiance, and the training of a deep learning model, the utilization of this method is most applicable to temporally persistent imaging of the same scene, for which a single model can be used to correct the spectral reflectance of all obtained scans. In this work, we select such an application to demonstrate the capabilities of this method at extracting the vegetation spectral reflectance from “real-world” sensor obtained spectral radiance, as described in the Case study section.

**Fig. 12:**
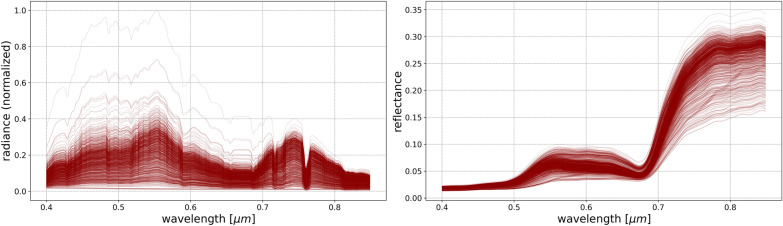
*Left:* the sensor-obtained uncorrected averaged and normalized spectral radiance of the vegetation canopy as identified in Fig. 2. *Right:* the associated solar and atmosphere corrected vegetation spectral reflectance as predicted by the Enc-Dec CNN

The ground-based, horizontally-aligned, south-facing HSI sensor in New York City captured approximately 1100 scans of the scene shown in Fig. 2 over the month of May 2016. The vegetation located approximately 1 km south of the sensor are identified, and their spectral radiance averaged to produce a single canopy spectral radiance per scan. The at-sensor obtained spectral radiances are shown in Fig. 12, and are the uncorrected photon flux measurements by the sensor, which are then min-max normalized and cut-off at 0.85 $\mu$m. Following the training of the Enc-Dec CNN using simulated spectral radiance that reflect the properties of the sensor, the normalized spectral radiance are used as input to the model, together with their known time and date of capture, and the output predicted vegetation spectral reflectance, corrected for atmospheric and solar effects, are shown for each of their associated spectral radiance in Fig. 12.

The model behaves as anticipated with “real” at-sensor spectral radiance data, where the predicted spectral reflectance all exhibit the known signature features that are unique to those of vegetation, including the chlorophyll bump and red edge. Given that we lack the knowledge of the exact solar and atmospheric parameters of each obtained scene, to provide further confidence in the ability of the model to extract the vegetation spectral reflectance from at-sensor spectral radiance, we compare the Enc-Dec CNN predicted output to those produced by another method that does not rely on the synchronous knowledge of such parameters. The Compound Ratio, as presented in more detail in [21], exploits the temporally static nature of the spectral reflectance of built structures to produce the relative change in the apparent reflectance of adjacent vegetation. Provided the assumptions that buildings have constant reflectivity over moderately short time spans ($R^B_{\lambda ,t} = R^B_{\lambda ,t=0}$), that the total irradiance incident on the target vegetation is identical to that incident on immediately adjacent buildings ($E^V_{\lambda ,t} = E^B_{\lambda ,t}$), and that atmospheric transmission between the target and sensor is identical to that between the building and sensor ($T^{V}_{\lambda ,t} = T^{B}_{\lambda ,t}$), the Compound Ratio of vegetation ($C^V_{\lambda ,t}$) at time *t* is calculated as:

4 $$\begin{aligned} C^V_{\lambda ,t} \equiv \frac{L^V_{\lambda ,t}/L^V_{\lambda ,t=0}}{L^B_{\lambda ,t}/L^B_{\lambda ,t=0}} = \frac{R^{*,V}_{\lambda ,t}}{R^{*,V}_{\lambda ,t=0}}. \end{aligned}$$

**Fig. 13:**
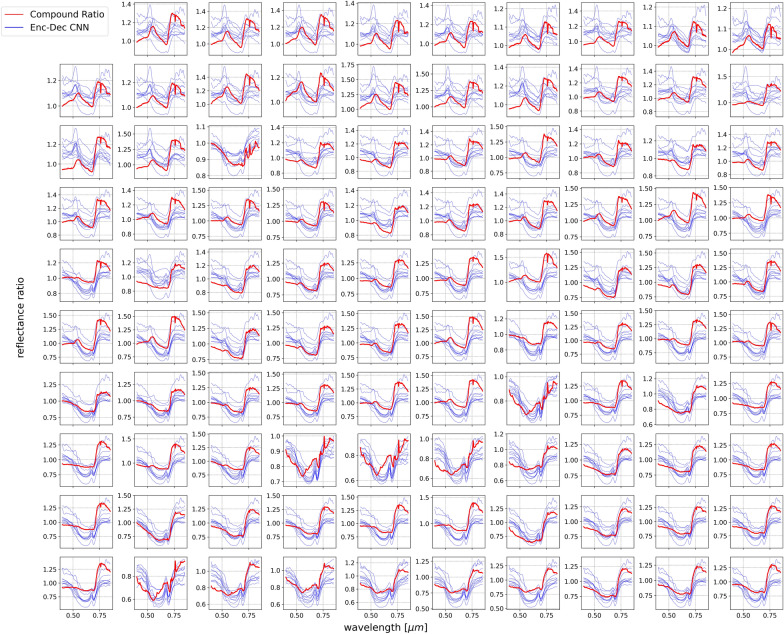
A randomly chosen sample of 99 scans showing the ratio of apparent reflectances of vegetation as computed using the Compound Ratio (*red*), together with the ratio of the atmospheric and solar corrected spectral reflectances as predicted by each of the 10 independently trained Enc-Dec CNN models (*blue*)

Using the spectral radiance of the buildings immediately adjacent to the vegetation, and considering the first obtained scan as having time $t=0$, we compute the Compound Ratio of the vegetation in each scene. In order for the output of the Enc-Dec CNN to be comparable to those from the Compound Ratio, 10 predicted spectral reflectances are produced for each scan from the 10 independently trained Enc-Dec CNN models, each prediction is then divided by the prediction from the same model for the initial scan at time $t=0$ to produce the relative change of spectral reflectance. Figure 13 shows 99 randomly chosen scans to compare the output from the Compound Ratio to those from the Enc-Dec CNN. Considering the variation in prediction between the 10 models to be the range of uncertainty in obtained spectral reflectance, the predicted ratio of spectral reflectance from the Enc-Dec CNN models are consistent with those produced by the Compound Ratio in nearly every scan.

## Discussion

The results of this study demonstrate that a framework that uses a time-dependent encoder-decoder convolutional neural network (Enc-Dec CNN), is capable of accurately obtaining the vegetation spectral reflectance from at-sensor hyperspectral radiance data with two temporal factors representing the time and day of image acquisition. The framework presented here can be compared with that of a “mixed” trained network with similar architecture, trained on 21 different materials, including 15 vegetation spectral reflectances and 6 opaque Lambertian gray bodies with constant reflectivities, to predict the spectral reflectance of 27 other materials [50], in contrast to the 1000 unique vegetation spectral reflectances generated using SCOPE in this work. The method introduced in [50] is aimed at efficiently maximizing the transferability of a single model to various materials and sensor acquisition elevation angles (30$^\circ$–90$^\circ$). Therefore, the mixed trained network is designed to output six normalized radiative transfer equation (RTE) components that can then be combined with the estimated temperature to generate the retrieved target reflectivity from input spectral radiance obtained under 13 different elevation angles. In contrast, our models are aimed at increasing the prediction accuracy of the spectral reflectance of vegetation from at-sensor spectral radiance obtained from a static ground-based hyperspectral sensor of known location, pointing, and angles. Consequently, we have developed a neural network that takes a single at-sensor spectral radiance as input and outputs a prediction of the wavelength-dependent deviation from the median simulated vegetation reflectance, $\Delta$reflectance, which is then trivially converted to the vegetation’s spectral reflectance. The mixed trained network of [50], used to predict the reflectance of 42 different materials, is reported to have an overall test dataset accuracy for predicting spectral reflectance of 95.7% [50]. The models we present here exhibit a test dataset $r^2$ of 92.0% (±1.0) with the $\Delta$reflectance, and 98.1% (±0.4) when converted to spectral reflectance. It is worth noting that while the proposed treatment in this work provides a significant enhancement to the accuracy of extracting reflectance, it does so at the expense of removing the flexibility to handle multiple materials other than vegetation and radiance received under variable elevation angles.

In addition to the high $r^2$ showing the capability of this model at overall correctly predicting the vegetation spectral reflectance, the errors are evenly and compactly distributed around the actual values, for all $\Delta$reflectances and wavelengths. This shows that the model exhibits both high accuracy and high precision when predicting the $\Delta$reflectance from the at-sensor spectral radiance and two temporal factors. While qualitative examinations of the $\Delta$reflectance results yield little insights into the presence of systematic mispredictions, when converted to spectral reflectance, a clear imbalance in the magnitude of errors (when they occur) is apparent between long and short wavelengths. Despite the variance in spectral reflectance at longer wavelengths being generally lower than the variance at shorter wavelengths, errors in prediction of reflectance are significantly larger at wavelengths $>0.75\mu$m. This imbalance can be partially attributed to the greater amplitude of the spectral reflectance in the infrared relative to the reflectance in the visible spectrum, which is a well-known characteristic of vegetation spectral reflectance, amplifying the effect of lower relative variance. Nevertheless, it is important to note that these errors overall are relatively small and infrequent. The overall $r^2$ of the predicted and actual reflectance spectra is 98.1% (±0.4), with the vast majority of instances having $r^2=100\%$ and being qualitatively indistinguishable from the ground-truth. Furthermore, the greatest deviation of prediction from reality produces spectral reflectance with $r^2>96\%$, and the uncertainties for all instances rarely exceed 1%.

Provided that the spectral radiance is a combination of the vegetation spectral reflectance and solar and atmospheric effects, the process for learning to extract the $\Delta$reflectance from the at-sensor spectral radiance is equivalent to that for learning to identify the absorption spectrum produced by solar and atmospheric effects. The encoder in the neural network reduces the dimensionality of the input spectral radiance to minimize redundant information and identify the particular features that can correctly provide either the dimensionally reduced $\Delta$reflectance spectrum or the complementary solar and atmospheric effects spectrum. Therefore, the dimensionally reduced encoding of the spectral radiance must contain sufficient information regarding both the vegetation spectral reflectance, and the solar and atmospheric effects. As evident by the common use of vegetation indices, in carefully selected couplings, the reflectance in only two channels can carry sufficient and specific information regarding the health and status of vegetation. However, atmospheric effects on radiation, particularly those from the absorption of gases and molecules, occur in signature narrow bands that can quickly dilute the information if captured at sufficiently low spectral resolutions. Despite this, reducing the resolution of the simulated spectra, while proportionally adjusting the network’s hyperparameters, results in consistently high testing accuracy for spectra modeled with spectral resolutions ranging from FWHM of 1 nm (450 bands between 0.4–0.85 µm, or equivalently, 600 bands in VNIR 0.4–1.0 µm), down to 40 nm FWHM (29 bands between 0.4–0.85 µm, or equivalently, 40 bands in VNIR 0.4–1.0 µm). This result indicates that the relevant impacts of atmospheric absorption and attenuation on light traversing the atmosphere and reflecting off of vegetation remain equally and highly identifiable in the VNIR wavelength range with 40 channels, as it does with 600 channels. Spectra at lower resolutions are expected to begin to lose this information, but even at resolution as low as 60 nm FWHM with the equivalent of only 10 channels in VNIR (7 channels between 0.4–0.85 µm), results in maintaining sufficient information on atmospheric effects to produce $\Delta$reflectances with $r^2$ in the range of 80% for the test dataset. At resolutions of single digit channels in the VNIR, any information on the impacts of the atmosphere on the vegetation spectral reflectance are quickly diluted beyond recognition, resulting in the incapability of the model as it stands to correctly predict the spectral reflectance.

Separating the training (50%), testing (30%), and validation sets (20%), both for the 1000 simulated spectral reflectance and 1440 emission and transmission profiles, and combining the sets produces a total of 547,200 instances. This is significantly fewer than the 1,440,000 instances that could be generated if the spectral radiance calculation took place prior to separating the sets. However, by separating first, it can be guaranteed that the model does not encounter either the spectral reflectance or the solar and atmospheric profiles, of which the testing spectral radiance is composed, prior to testing. This provides confidence in the model’s testing metrics being truly reflective of the transferability of the model to predict spectral reflectances in atmospheres it has not previously encountered. Repeating this process a total of 10 times, each using separate and unique sets of training, validation, and testing instances from the others, shows the range and consistency of the performance of the model when using 10% of the total available number of instances. When the number of available training examples is further reduced, we find a measurable reduction in the model’s accuracy, which indicates that the model may be able to provide greater accuracy if trained and tested on the full sample of instances rather than as an ensemble. Naturally, it can also be expected that such a treatment may also result in greater risk of overfitting.

Applying this framework to field obtained ground-based remote hyperspectral radiance validates that the model behaves as expected in terms of producing vegetation spectral reflectances that contain the known signature features of the interaction of plants with light in the VNIR wavelength range. Training the Enc-Dec CNN on synthetic spectral radiance by using the known parameters of the sensor, including location, orientation, and optics, which resemble the true normalized photon flux radiance obtained by the sensor, together with the known date and time of image acquisition results in the model producing corrected spectral reflectance that demonstrate the general validity of this framework. Comparing the ratio of predicted spectral reflectance to those produced using the Compound Ratio approach, which exploits the temporal independence of nearby buildings’ albedo, shows that both methods provide results within the bounds of the uncertainty ranges. While it is not possible to extract the true spectral reflectance of vegetation from the spectral radiance without explicit, accurate, and synchronous knowledge of the solar and atmospheric parameters of each obtained image, the agreement between the two methods demonstrates the merit and utility of the proposed Enc-Dec CNN framework in a real world application.

Sources of uncertainty in the spectral reflectance prediction can be attributed to the difficulty in producing perfect analogues for real at-sensor spectral radiance. For example, despite the ability of SCOPE to produce the spectral reflectance of the vegetation canopy mixed with the reflectance of the soil at the bottom layer, the spatial resolution of the sensor together with its horizontal orientation and the presence of various materials in an urban setting result in numerous potential spectral mixing scenarios that could not be considered in this framework. Furthermore, SCOPE assumes a 1-dimensional (vertical) turbid medium canopy, and is thus less adept at simulating discontinuous or open canopies. Therefore, aside from the apparent process of increasing the abundance of training instances, model performance can be enhanced with other means that may address the aforementioned sources of uncertainty. Given that the adjustment of the model towards increased specialization in target material results in increased model performance, as evident in this study, further restriction of the scope of materials addressed by the model to one or a few known target species may result in increased prediction accuracy. While model specialization may provide prediction accuracy enhancement, it provides it at the cost requiring the remodeling of spectra for each specific configuration rather than a generalized approach. Similarly, this model may benefit in increased prediction accuracy with fewer synthetic instances needed if performed with the knowledge of some atmospheric parameters or vegetation characteristics to reduce the total number of possible permutations.

## Conclusions

In this work, we propose a framework for the extraction of vegetation spectral reflectance in hyperspectral remote ground-based images in the presence of unknown atmospheric and solar effects. This method relies on using a time-dependent encoder-decoder convolutional neural network (Enc-Dec CNN), trained and tested using simulated at-sensor spectral radiance produced from combining 1440 unique simulated solar and atmospheric profiles, and 1000 different spectral reflectances of vegetation with various health indicator values, both produced using the specialized radiative transfer modeling codes SMARTS2 and SCOPE, respectively. The total dataset is separated into 10 subsets, each divided into independent training, testing, and validation sets, where instances between sets differ in both spectral reflectance and solar and atmospheric profiles, and 10 separate models are independently trained, validated, and tested, each on one of the synthetic sample subsets. These treatments to the simulated dataset are performed to provide confidence ranges for the performance metrics of the model, and ensure that the model is being tested for its transferability to instances it has not previously encountered in the training.

For spectra simulated with 600 channels in the VNIR wavelength range with a FWHM of 1 nm, the proposed method is capable of predicting the deviation of the spectral reflectance from the median simulated spectrum ($\Delta$reflectance) with a testing $r^2$ of 92.0% (±1.0). When converted to the spectral reflectance, the model results in an $r^2$ of 98.1% (±0.4), where the vast majority of predicted spectral reflectances show $r^2=100\%$, and, when present, errors in prediction are mainly confined to wavelengths $>0.75\mu$m. Furthermore, the high performance of this method at predicting $\Delta$reflectance is consistently sustained at $r^2>90\%$ for spectral radiances captured at reduced resolution, down to a FWHM of 40 nm with 40 channels in VNIR. This method remains viable, with $r^2 > 80\%$ down to resolutions of 10 channels in VNIR with FWHM of 60 nm, beyond which the information contained in the spectral radiances regarding atmospheric composition and vegetation spectral reflectance is quickly diluted and lost.

Therefore, this proposed method is shown to be highly adept at accurate and precise solar and atmospheric correction of vegetation spectral reflectance from remote ground-based sensor-measured spectral radiance without explicit knowledge of atmospheric compositions and conditions. While focusing a network on solely predicting the spectral reflectance of vegetation,

rather than a variety of different material types, comes at the cost of reduced transferability of the trained model to predict the spectral reflectance of other materials, our results indicate that generating large sets of training instances that adequately encompass true vegetation spectral reflectance yields significant improvements in prediction accuracy, suggesting that generating the associated tailored simulations and model retraining may potentially result in similar model improvements for other materials as well.

## Data Availability

The datasets used and/or analyzed during the current study are available from the corresponding author on reasonable request.
